# Neural Entrainment Meets Behavior: The Stability Index as a Neural Outcome Measure of Auditory-Motor Coupling

**DOI:** 10.3389/fnhum.2021.668918

**Published:** 2021-06-09

**Authors:** Mattia Rosso, Marc Leman, Lousin Moumdjian

**Affiliations:** ^1^Institute of Psychoacoustics and Electronic Music (IPEM), Faculty of Arts and Philosophy, Ghent University, Ghent, Belgium; ^2^UMSC Hasselt-Pelt, Limburg, Belgium; ^3^REVAL Rehabilitation Research Center, Faculty of Rehabilitation Sciences, Limburg, Belgium

**Keywords:** auditory-motor coupling, entrainment, synchronization, instantaneous frequency, eigendecomposition, finger-tapping, stability index, EEG

## Abstract

Understanding rhythmic behavior in the context of coupled auditory and motor systems has been of interest to neurological rehabilitation, in particular, to facilitate walking. Recent work based on behavioral measures revealed an entrainment effect of auditory rhythms on motor rhythms. In this study, we propose a method to compute the neural component of such a process from an electroencephalographic (EEG) signal. A simple auditory-motor synchronization paradigm was used, where 28 healthy participants were instructed to synchronize their finger-tapping with a metronome. The computation of the neural outcome measure was carried out in two blocks. In the first block, we used Generalized Eigendecomposition (GED) to reduce the data dimensionality to the component which maximally entrained to the metronome frequency. The scalp topography pointed at brain activity over contralateral sensorimotor regions. In the second block, we computed instantaneous frequency from the analytic signal of the extracted component. This returned a time-varying measure of frequency fluctuations, whose standard deviation provided our “*stability index*” as a neural outcome measure of auditory-motor coupling. Finally, the proposed neural measure was validated by conducting a correlation analysis with a set of behavioral outcomes from the synchronization task: resultant vector length, relative phase angle, mean asynchrony, and tempo matching. Significant moderate negative correlations were found with the first three measures, suggesting that the stability index provided a quantifiable neural outcome measure of entrainment, with selectivity towards phase-correction mechanisms. We address further adoption of the proposed approach, especially with populations where sensorimotor abilities are compromised by an underlying pathological condition. The impact of using stability index can potentially be used as an outcome measure to assess rehabilitation protocols, and possibly provide further insight into neuropathological models of auditory-motor coupling.

## Introduction

Auditory stimuli such as music or metronomes can entrain human movement, and this phenomenon can be used for neurological rehabilitation purposes. Particularly, evidence has been established that auditory stimuli can facilitate walking in persons with Parkinson’s disease (Ghai et al., 2018; De Bartolo et al., 2020), stroke (Yoo and Kim, 2016; Hutchinson et al., 2020), and multiple sclerosis (Moumdjian et al., 2019b). Auditory stimuli convey temporal structures that serve as affordances for the motor system to interact with (Leman, 2016). In our previous work, we showed that auditory rhythms can entrain a person’s motor rhythms, thus affecting abilities for walking. The underlying mechanism can be explained in terms of sensorimotor phase-locking, prediction error minimization, and/or dynamical interactions (Phillips-Silver et al., 2010; Leman, 2016). The outcome of an entrainment process is typically a more stable state of synchronization (Phillips-Silver et al., 2010; Moens et al., 2014; Leman, 2016). So far, the entrainment effect has been quantified by means of behavioral outcome measures, in particular temporal outcomes of the rhythmic auditory-motor coupling (Moumdjian et al., 2018), which contributed to a better understanding of underlying mechanisms as a result of the interaction (Moumdjian et al., 2019c, 2020), and to the development of task-oriented training tools for walking in persons with the neurological disease of multiple sclerosis (Moumdjian et al., 2019a, b).

Part of the variability in entrainment can be attributed to individual synchronization abilities. When presented with auditory stimuli and asked to walk to them, there are participants who spontaneously synchronize, and others who do not. This ability is not only limited to neurological populations (Moumdjian et al., 2019c), but also holds true for healthy participants (Van Dyck et al., 2015) where the percentage of spontaneous synchronizers vs. non-synchronizers is about 50% −50%. A number of factors contribute to the tendency to rhythmically entrain and synchronize (Wilson and Cook, 2016). The first factor is temporal perception and prediction. Studies on Parkinson’s disease have concluded that those participants with higher perceptual sensorimotor synchronization abilities, quantified by behavioral sensorimotor tapping tasks involving finger tapping (Dalla Bella et al., 2017b), had a better outcome on their walking parameters after being subjected to walk to auditory stimuli (Dalla Bella et al., 2017a). The second factor is motor (e.g., physical capacity) and/or cognitive (e.g., attentive and pre-attentive) functions. For example, studies comparing spontaneous and instructed synchronization of walking (Leow et al., 2018; Moumdjian et al., 2019c) and running (Van Dyck et al., 2020) to music have been conclusive that explicit instructions to synchronize resulted in a higher synchronization tendency, as compared to spontaneous synchronization. The former study also noted this difference across different motor thresholds which was provided as a result of walking to different tempi, starting from the natural comfortable tempo and up to + 10%, in increments of 2% (Moumdjian et al., 2019b, c).

Up to now, most studies on neurological populations, investigating entrainment and synchronization during walking tasks, are based on empirical evidence using behavioral outcomes (Moumdjian et al., 2018). However, we believe that the development of complementary neurological outcomes could offer a further understanding of entrainment and synchronization, potentially leading to the development of more individualized and more fine-tuned rehabilitation approaches.

**Figure 1:**
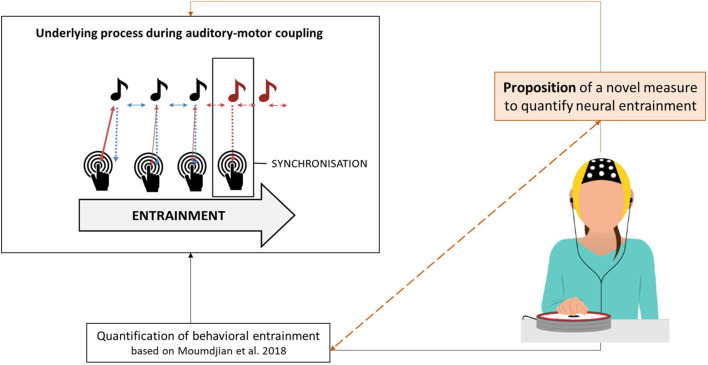
Graphical illustration of the study’s rationale and the proposed contribution to the current state of the art.

The present study, therefore, aims at quantifying a neural outcome measure of entrainment and synchronization in combination with behavioral outcomes. We propose the use of electroencephalography (EEG) as a method to measure neural entrainment of the motor system to rhythmic stimuli. The novel outcome measure is based on a finger tapping task (Bavassi et al., 2013; McPherson et al., 2018; Lopez and Laje, 2019). Figure 1 shows a graphical illustration of this study’s rationale and proposed contribution to the current state of the art.

Our approach is based on Steady-State Evoked Potentials (SSEPs) (Vialatte et al., 2010; Norcia et al., 2015). Given a steady periodic stimulation, a series of subsequent evoked responses are elicited in the electrical brain activity, generating a periodic pattern of transients in the EEG signal. By transforming the signal to the frequency domain by means of Fast Fourier Transform (FFT), it can be observed that the EEG spectrum is dominated by a prominent peak at the stimulation frequency and its harmonics. Upon exposure to rhythmic auditory stimuli, patterns emerge in brain activity and match the dominant spectral features of the stimulation. Studies show that neural entrainment can be measured at different hierarchical levels of the stimulus temporal structure, or of its representation (Nozaradan et al., 2011). As the sound envelope of a musical stimulus exhibits a periodic low-frequency amplitude modulation in correspondence with the beat, it is possible to observe a match between the beat-related harmonics of the EEG spectrum and the sound spectrum (Lenc et al., 2018). However, the observed entrained components are not always entirely driven by sensory stimulation. In fact, given the same energy in the stimulus, SSEP amplitude is modulated by attention (Andersen et al., 2011; Kashiwase et al., 2012), internal representation of metric structure (Nozaradan et al., 2012), sensorimotor integration (Nozaradan et al., 2015) and interpersonal coordination (Varlet et al., 2020).

The SSEP technique is relatively straightforward in modeling bottom-up and top-down components of rhythm perception in terms of Fourier coefficients. However, in order to link behavioral entrainment to a neural outcome measure, we believe that the signal phase should not be left out of the picture. Rajendran and Schnupp (2019) showed that shuffling the phase of a signal resulted in drastic differences in its time domain representation, whereas it remained invariant in the frequency domain. Although the analysis of peak amplitudes or *z*-scoress in a static spectrum might convey information about the outcome of neural entrainment, it is arguably insensitive to its dynamics in the time domain. One should consider that oscillatory processes in the brain are hardly stationary (Cohen, 2017) and the very definition of entrainment implies that an oscillator dynamically changes its frequency in order to achieve stable synchronization. This is precisely the phenomenon we intend to capture. Therefore, in order to quantify neural entrainment of rhythmic stimuli, we argue in favor of a time-varying measure based on the phase of the neural entrained component.

With this study, we progress beyond the state of the art in the research on neural entrainment by optimizing the calculation of a neural outcome measure of auditory-motor coupling. We argue that such a measure can be used together with its behavioral counterparts. In combination, both the behavioral and neurological measures may unveil a further layer of the underlying mechanisms of the rich dynamical processes during motor and auditory interactions. Our first aim is to extract from the EEG signal the component which is maximally entrained to a periodic stimulus. For that, we compute a *stability index* to quantify frequency fluctuations over time. Our second aim is to validate the proposed index with a set of quantified behavioral outcome measures of auditory-motor coupling and entrainment (Moumdjian et al., 2018). In an auditory-motor coupling task, healthy participants were instructed to tap their index finger synchronizing to an auditory metronome (as illustrated in Figure 1). We hypothesized that our stability index would significantly correlate with the behavioral measures of entrainment. Specifically, a stable behavioral performance is expected to correlate with a stable entrained component, whereas a poor performance would result in wider frequency fluctuations over time.

## Materials and Methods

### Participants

Twenty-eight (*N* = 28) right-handed participants took part in the study (18 females, 10 males; mean age = 29.07 years, standard deviation = 5.73 years). None of them had a history of neurological, major medical, or psychiatric disorders. All of them declared not to be professional musicians upon recruitment, although some of them had musical experience. Handedness was assessed by means of the Edinburgh Handedness Inventory (Oldfield, 1971). The experiment was approved by the Ethics Committee of Ghent University (Faculty of Arts and Philosophy) and informed written consent was obtained from each participant, who received a 15€ coupon as economic compensation for their participation.

### Experimental Procedure

The experimental task consisted of a tapping synchronization paradigm, in a sitting position. Participants were provided with a custom-made pad containing piezo sensors to detect tapping onsets, and were instructed to tap their right index finger along with the assigned metronome during 390 seconds. During the task, participants were sitting on a comfortable chair equipped with armrests, so that their elbow could lay in a fixed position. Tapping movements were limited to wrist flexion in order to prevent movement-artifacts contamination of the EEG signal. Participants were monitored online and video-recorded by means of a USB camera to verify their compliance with the instructions. The importance of avoiding head and trunk movements was stressed.

### Auditory Stimuli

Participants were presented with the stimuli *via* DefenderShield^®^ air-tube earplugs. Ableton Live 10^®^ was adopted as software for the metronome stimuli presentation. A periodic auditory cue was presented at a rate of 100 BPM to half of the participants, and 98.5 BPM to the other half (1.67 Hz and 1.64 Hz, respectively). The reason for such a minimal gap lies in the rationale of a larger experimental design in which the recordings were performed (Rosso et al., under review).

### Behavioral Data Acquisition

Finger tapping onsets were recorded with a Teensy 3.2 microcontroller, operating as serial/MIDI hub in the setting. On the one hand, it received an analog input from piezo sensors inside the pads and printed on the serial port of the stimulation computer a timestamp each time a finger-tap pushed the signal above a resting threshold. The threshold was conservative enough to prevent false positives due to signal bouncing. Every time a metronome beat onset was presented to a participant, a MIDI message was sent to the Teensy to log its timestamp on the serial port. All timestamps were rounded to 1 ms resolution, which corresponds to 1 kHz sampling rate. The same device triggered the start of the EEG recording by sending a TTL trigger *via* a BNC connection.

### Outcome Measures

Behavioral data and neurophysiological data were measured. These are outlined below:

#### Behavioral Data

The timestamps of finger-tapping and metronome beat onsets were imported in Matlab^®^ and used to calculate a set of behavioral outcome measures of auditory-motor coupling and entrainment (Moumdjian et al., 2018). Before doing so, we removed the finger-tapping onsets following the previous one by less than 350 ms, as false positives could occasionally be recorded when a participant pushed the pad for too long or accidentally laid the hand on it. On average, 0.4 false positives were removed for every participant (standard deviation = 0.8). From the finger-tapping and metronome beat onsets time series, we calculated the following measures: relative phase angle, resultant vector length, mean asynchrony, and tempo matching. Below, details of the measures and the formulae used calculate these measures are outlined (Moens et al., 2014; Moumdjian et al., 2018):

#### Relative Phase Angle

This is an error measure of synchronization based on the phase difference between two oscillators (i.e., the participant tapping and the metronome beat onsets).

$$\varphi =360*\left(\frac{T_{n}-B_{n}}{B_{\left(n+1\right)}-B_{n}}\right)$$

Where *T_n_* is the participant’s tap onset *n* and *B_n_* is the onset of the closest metronome beat. A negative angle indicates that the participant is tapping ahead of the metronome beat, while a positive angle indicates that the participant’s tap is lagging behind the metronome beat. Alternatively, following recent work on modeling participants and periodic cues of systems of coupled oscillators in finger-tapping studies (Heggli et al., 2019), we processed the phase time series for participants and metronomes by interpolating the onsets as a ramp wave, wrapped from 0 to 2π radians at 1 kHz sampling rate. Provided with an estimate of the oscillators’ positions on their cycle with a temporal resolution of 1 ms, we subtracted each participant’s phase time series from the respective metronome. Finally, the CircStats toolbox (Berens, 2009) for Matlab^®^ was used to calculate the mean angle from the resulting relative phase time series (in radians).

#### Resultant Vector Length

This expresses the stability of the relative phase angles over time. A unimodal distribution implies a high resultant vector length, whereas uniform and bipolar distributions result in a low resultant vector length. The measure was processed with the CircStats toolbox (Berens, 2009), using the relative phase time series as input. The measure ranges from 0 to 1, where 1 indicates perfect synchronization over time at a given relative phase angle and is calculated as follows:

$$R=\left|\frac{1}{N}\sum_{n-1}^{N}e^{i\phi T_{n}}\right|$$

#### Mean Asynchrony

This consists of the mean difference between the participant’s tap onsets and the respective closest metronome’s beat onset expressed in milliseconds.

$$Mean\;asynchrony=\frac{1}{N}\sum_{n-1}^{N}T_{n}-B_{n}$$

#### Tempo Matching Accuracy

This indicates to what extent the overall tempo of the participant’s tapping matches with the tempo of the metronome beats, based on inter-onset-intervals (IOIs). Inter-beat deviation (IBD) is calculated as the mean difference of a subject’s IOIs with respect to the inter-beat-intervals.

$$IDB=\frac{1}{N}\sum_{n-2}^{N}\frac{\left(B_{n}-B_{\left(n-1\right)}\right)-\left(T_{n}-T_{\left(n-1\right)}\right)}{B_{n}-B_{\left(n-1\right)}}$$

### Neurophysiological Data

**Figure 2:**
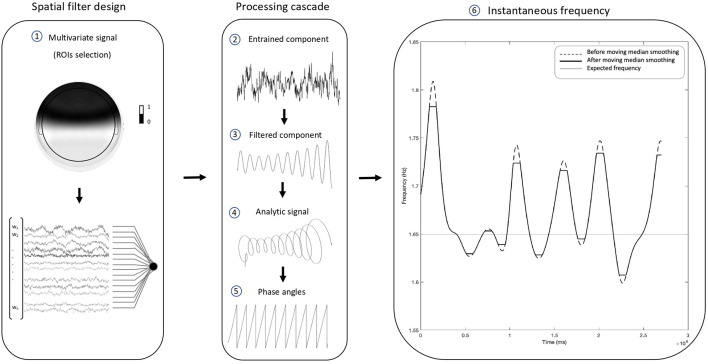
Electroencephalographic (EEG) processing pipeline. The present pipeline illustrates the steps through which the proposed stability index was computed. Following the pre-processing, generalized eigendecomposition (GED) was performed on a broad set of regions of interest (ROIs). The vector of weights w associated with the highest eigenvalue was used as a spatial filter. By multiplying the data from the 37 channels behind the frontocentral line (1), we produced a single time series. The weights of the excluded channels were set to 0. The resulting “entrained component” (2) went through a cascade of computational steps: first, it was narrow-band filtered with a Gaussian filter centered at the stimulus frequency, in order to extract reliable phase time series unaffected by broad-band components (center = 1.65 Hz; width at half-maximum = 0.3 Hz). The “filtered component” (3) was then Hilbert-transformed to produce the “analytic signal” (4), from which we computed the “phase angles” time series (5). Finally, the phase was unwrapped, its first derivative was used to compute the “instantaneous frequency” (6), and a sliding moving median was applied in order to level out eventual artifactual peaks. The plot shows how the pipeline results in a time-varying measure of frequency over time, which fluctuates around the stimulation frequency (i.e., the thin horizontal line intercepting the y-axis at 1.65 Hz). The standard deviation of the instantaneous frequency provides a global measure of the stability of the entrained component for a given time window, which in our case was the whole duration of the task. We named such a global measure “stability index”, for it equals 0 in the case of a flat horizontal line. Such a scenario would be observed in the ideal case of a perfectly stable component oscillating like a simple sine wave.

In order to compute our proposed outcome measure of neural entrainment, the following EEG processing pipeline was conducted. It consists of signal pre-processing, extraction of the entrained component *via* generalized eigendecomposition, and the computation of the stability index. The workflow is summarized in Figure 2.

#### Data Acquisition

Participants were equipped with a 64-channel waveguard^TM^ original EEG headset (10-10 system, with Ag/AgCl electrodes). Data were recorded with an ANT-Neuro *eego^TM^mylab* system at 1 kHz sampling rate. Impedances were monitored in the *eego^TM^* software environment and kept below 20 kΩ. In comparison with stricter thresholds (e.g., 5 kΩ or 10 kΩ), the choice made it feasible to maximize the homogeneity of impedance levels across electrodes, and in turn, optimize the covariance matrices used in our source separation. A referential montage was adopted, with “CPz” as the reference electrode.

#### Pre-processing

Pre-processing was carried out with a pipeline integrating functions from the *Fieldtrip toolbox* (Oostenveld et al., 2011) for Matlab (MathWorks Inc., USA). Bad channels were identified by means of visual inspection of raw time series and variance distribution across channels. The recordings were re-referenced to the average of all the electrodes after channel rejection, to avoid noise leakage into the average. A high-pass Butterworth filter with 1 Hz cut-off was applied to the raw recordings to remove slow drifts. We preferred to choose this conservative threshold, given that occasional head movements and sweat potentials are more likely to occur over a long continuous recording. A low-pass Butterworth filter with 45 Hz cut-off was applied to remove high-frequency muscular activity. A notch filter centered at 50 Hz was applied to remove power-line noise up to the 3rd harmonic.

Independent component analysis (ICA) was conducted on full rank data to remove blinks and eye-movement artifacts, by means of visual inspection of topographical maps and time series of component activation. For this purpose, we ran the “runica” algorithm as implemented in Fieldtrip, excluding the reference “CPz” and the bad channels time series from the input matrix. Only those components which exhibited the stereotyped frontal distribution generated by blinks and lateral eye movements were removed. Although other artifactual sources could have been identified, we limited the selection to a few unambiguous components for the sake of replicability. A minimum of one and a maximum of three components were removed for every participant. The dataset was inspected prior to ICA decomposition and following ICA back-projection. Special attention was given to the electrodes where the activation of the artifactual component was maximal, namely the F, AF, and Fp clusters. Rejected bad channels were finally reconstructed after artifact removal, by computing a weighted average of all neighbors as implemented in Fieldtrip.

Recordings were treated as a continuous experimental run, without segmentation in epochs. This implies that no “bad trials“ were removed. Further in this section, we will present how we dealt with transient bursts of artifactual activity in the continuous recording.

#### Generalized Eigendecomposition (GED)

In order to avoid channel selection bias while optimizing the signal-to-noise ratio between the entrained component and the broadband neural activity, we applied GED as first described in the context of source separation for rhythmic entrainment (Cohen and Gulbinaite, 2017). The technique consists of a spatial filter to reduce the multivariate dataset to one dimension, guided by some criteria: in this case, it was attunement to the stimulation frequency. This was achieved by computing the weighted average of a set of channels, where the vector of weights *W* was calculated by solving the following eigenequation:

$$R^{-1}SW=\Lambda W$$

where S is the covariance matrix calculated from the narrow-band filtered signal; R is the reference covariance matrix calculated from the broad-band signal; Λ is a set of eigenvalues. GED identifies eigenvectors W that best separate the signal (“S”) covariance from the reference (“R”) covariance matrix. The eigenvector associated with the largest eigenvalue is taken as a spatial filter. That eigenvector is then used to multiply the raw channel data to produce the single time series of our target entrained component. In the present work, a subset of 37 channels located behind the frontocentral “FC” line (mastoids excluded) was selected. By doing so, we intended to constrain the source separation and target a sensorimotor component entrained to the auditory stimulus. The excluded channels form the cluster which is typically expected from a purely auditory response at the scalp level (Nozaradan et al., 2011, 2015). The regions of interest (ROIs) selection is visually illustrated in Figure 2.

Given we were explicitly looking for frequency fluctuations, our narrow-band filter needed to be large enough in order to leave room for fluctuations around the entrained frequency. We designed our filter as a Gaussian function in the frequency domain, with the center at 1.654 Hz and a width of 0.3 Hz at half of the maximum gain. The center corresponds to the average of the two metronome frequencies. Given that the minimal difference across frequencies (1.667 Hz and 1.641 Hz) was tested to be negligible, we opted to design one single filter centered on their average. Such parameter represents an optimal trade-off in our application since it allows for fluctuations around the center frequency without overlapping with the high-pass band filter (cut-off = 1 Hz). We then filtered the signal on the whole subset of 37 channels by performing element-wise multiplication between the signal spectrum and the filter kernel. The resulting spectrum was eventually transformed with inverse Fast Fourier Transform back in the time domain. The frequency-domain representation of the filter kernel is provided in Supplementary Figure 1, along with additional information in the figure’s description.

The reference R covariance matrix was here computed from the broadband multivariate signal. Our choice differs from the approach originally proposed by Cohen and Gulbinaite (2017) in that they propose a use-case for higher frequency ranges, which allows us to average the R matrices computed from two narrow-band Gaussian flankers neighboring the central filter on both sides. Their rationale was to minimize the contribution of intrinsic non-task-related rhythms in frequency ranges far from the one of interest, while avoiding bias from upper and lower frequency neighbors. Given that we were dealing with low frequencies (<2 Hz), it was not desirable for us to narrow-band filter the signal in a lower flanker, as we would have reached below the high-pass filter cut-off at 1 Hz (see Supplementary Figure 1). Therefore, if we adopted flankers, we would have had a bias to the right side of the spectrum.

In order to compute the respective covariance matrices from the broad-and narrow-band signals, we used the onset timing of the finger-taps performed by the participant to define time windows from −100 ms to 500 ms around the events. The approach provided us with a considerable number of covariance matrices for each recording (645 finger-taps were expected on average), such that we could remove the ones whose Euclidean distance from the grand-average covariance matrix exceeded the 2.23 *z*-scoress (i.e., corresponding to a probability of 0.013). The grand-average S and R covariance matrices were then calculated free from the occasional burst of artifactual activity over the long recording, compensating from the impossibility of performing a procedure of trial-removal during the pre-processing.

**Figure 3:**
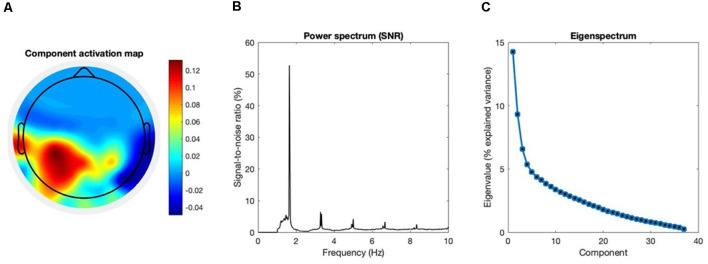
Group-level assessment of the source separation. The following criteria were used to assess the quality of our source separation *via* generalized eigendecomposition (GED). **(A)** Topography. The grand-average coefficients of activation are shown in the topographic plot: maximal activation was recorded at the left centroparietal “CP” cluster and at left temporal electrodes (“T7” and “TP7”). It should be noted that we explicitly excluded from the spatial filter the channels located beyond the frontocentral line, for we intended to maximize an entrained response related to sensorimotor processing in the context of the task. **(B)** SNR spectrum. The grand-average power spectrum is represented here in the percentage signal-to-noise ratio between each data point and the mean power in the neighboring bins (0.5 Hz), in order to remove the physiological 1/f component of the spectrum (Freeman et al., 2003). **(C)** Eigenspectrum.The grand-average eigenvalues sorted in descending order exhibit a steep exponential decay. The vector of weights *w* used for our spatial filter is the one associated with the highest eigenvalue λ. Before averaging, eigenvalues were normalized and expressed as percentage of explained variance. All grand-averages were computed on the whole sample of participants (N = 28).

The quality of our GED application was assessed by inspecting the eigenspectrum, the topographical activation map, and the power spectrum of the extracted component (see Figure 3).

#### Stability Index

Once the entrained component was computed, we applied on it the same Gaussian filter (center at 1.654 Hz and 0.3 Hz width at half maximum) in order to extract reliable phase time series from the analytic signal. We calculated the analytic signal with the Hilbert transform and computed the instantaneous frequency time series from the first derivative of the unwrapped phase angles time series as indicated in Cohen (2014). The instantaneous frequency of a dynamical oscillating system can be defined as the change in the phase per unit time (Boashash, 1992). The derivative can then be converted to Hz applying the following formula:

$$Hz_{t}=\frac{s(\phi_{t}-\phi_{t-1})}{2\pi }$$

where *s* indicates the data sampling rate and Φ*_t_* indicates the (unwrapped) phase angle at time *t*. A sliding moving median with a window width of 400 samples was used to smooth the instantaneous frequency time series, to remove occasional extreme bursts due to artifactual activity distorting phase time series. Finally, we calculated the standard deviation of instantaneous frequency over the whole task as a global measure of frequency stability over time, which we named the “stability index”. A high standard deviation is thus indicative of wide instantaneous frequency fluctuations, and less overall stability of the entrained component. A standard deviation equal to 0 indicates a perfectly stable component, with the instantaneous frequency being a flat line at the constant value of the stimulus frequency.

### Statistical Analysis

In order to validate our neural outcome measure, we calculated the Spearman coefficient for the correlation between the stability index and the four behavioral outcome measures (Moumdjian et al., 2018) reported above. This technique assesses the strength and significance of monotonic relationships between variables, regardless of its linearity. The Spearman correlation coefficient computed on continuous variables is the equivalent of the Pearson correlation coefficient computed on their ranks: it is exempt from the assumption of normal distribution of the pair of variables and robust to outliers and scaling effects. The following classification was used to categorize the correlation (Hinkle et al., 2002): 0.00–0.30 “negligible correlation”, 0.30–0.50 “low correlation”, 0.50–0.070 “moderate correlation”, 0.70–0.90 “high correlation”, 0.90–0.100 “very high correlation”.

## Results

### Behavioral Outcome Measures

On a group level, we report that participants anticipated their tapping onsets relative to the beat, with a mean relative phase angle of −1.050 ± 0.681 radians and a mean asynchrony of −77.472 ± 40.603 ms. In addition, on a group level, they obtained a consistent synchronization with a relative vector length of 0.831 ± 0.156, and a consistent period measured by the inter-beat deviation of −0.001 ± 0.01. The individual participant behavioral results of these outcomes are reported in Table 1.

**Table 1:** The results of neural and behavioral outcome measures of entrainment per participant.

Participant ID	Neural outcome measure of entrainment	Behavioral outcome measures of entrainment			
	Stability Index (frequency fluctuation—std)	Relative phase angle (radians)	Resultant vector length (0-1)	Mean asynchrony (ms)	Inter-beat deviation (ratio)
1	0.033	−0.387	0.958	-37.259	0.000
2	0.088	−2.332	0.647	-76.046	-0.003
3	0.023	−0.436	0.952	-41.668	-0.006
4	0.024	−0.464	0.975	-44.403	0.000
5	0.057	0.078	0.939	7.412	-0.008
6	0.138	−1.503	0.711	-122.858	0.008
7	0.077	−0.867	0.787	-83.514	-0.002
8	0.060	−0.772	0.803	-76.068	-0.003
9	0.056	−2.543	0.298	-26.583	0.042
10	0.061	−1.337	0.629	-114.613	0.001
11	0.037	−0.547	0.898	-53.906	0.000
12	0.086	−1.349	0.817	-120.805	0.000
13	0.070	−2.358	0.500	-41.690	-0.002
14	0.088	−1.273	0.800	-122.573	-0.024
15	0.045	−0.790	0.872	-74.723	0.001
16	0.125	−1.364	0.900	-130.310	0.001
17	0.051	−1.621	0.843	-152.019	0.003
18	0.028	−0.590	0.955	-57.088	-0.001
19	0.030	−0.744	0.943	-72.028	-0.009
20	0.074	−2.285	0.795	-156.864	0.001
21	0.076	−0.301	0.820	-27.093	-0.020
22	0.050	−0.574	0.943	-55.641	0.001
23	0.069	−1.223	0.824	-118.943	-0.004
24	0.032	−0.937	0.871	-90.031	-0.001
25	0.030	−0.674	0.960	-65.188	-0.001
26	0.041	−0.519	0.963	-50.530	-0.002
27	0.117	−1.018	0.931	-99.138	0.001
28	0.062	−0.670	0.942	-65.033	-0.004

*Abbreviations: std, standard deviation; ms, milliseconds*.

### Neural Outcome Measures

#### Generalized Eigendecomposition

The source separation successfully extracted the entrained neural component of interest, as assessed by its spectral features and its topographical map of activation. The component associated with the higher eigenvalue was selected for our analyses. Additionally, we verified that the component associated with the second eigenvalue was not related to the behavioral performance. More details about the second component are provided in the Supplementary Figures 2, 3. A detailed profile of the first component is provided in Figure 3, and its functional meaning will be further discussed in the next section.

#### Stability Index

The stability index was computed as the standard deviation of the component’s instantaneous frequency, as described in the “Materials and Methods” section. On a group level, the stability index resulted in 0.062 ± 0.030 Hz. A stability index of 0 indicates a perfectly stable component, without any frequency fluctuation over time. The individual participant results of the stability index are reported in Table 1.

### Correlation Analysis

**Figure 4:**
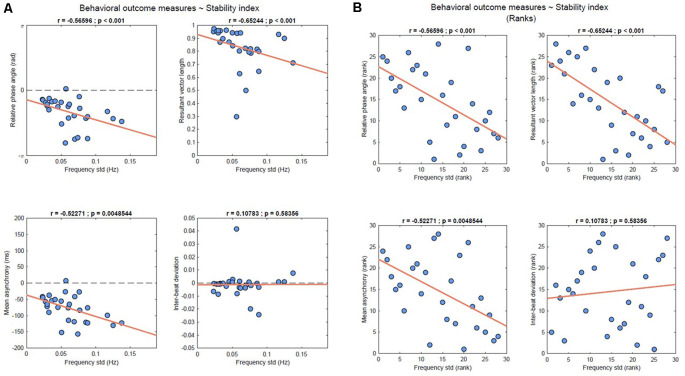
**(A)** Results of the Spearman’s correlation analysis between the behavioral outcome measures and stability index of all study participants. Data are represented on the original scale. **(B)** Correlations between the ranks for the behavioral outcome measures and stability index of all study participants.

As shown in Figure 4, Spearman correlation between the behavioral outcome measures of entrainment and the stability index revealed significant moderate negative correlations for relative phase angle, resultant vector length and mean asynchrony (*r* = −0.566, *p* < 0.001; *r* = −0.652, *p* < 0.001; *r* = −0.523, *p* = 0.005, respectively). A non-significant negligible correlation was found for the inter-beat deviation (*r* = 0.107, *p* = 0.583).

## Discussion

The main contribution of the present work is methodological, motivated by the need to compute a neural outcome measure of neural entrainment in the context of auditory-motor coupling and prospectively applying auditory-motor coupling paradigms for the purpose of neurological rehabilitation. We proposed a novel processing pipeline to compute the stability index, and validated this neural outcome measure by testing its correlation with a set of behavioral outcome measures in the context of a finger-tapping task.

Behaviorally, participants exhibited the mean negative asynchrony typically reported in finger-tapping synchronization tasks performed by healthy participants (Aschersleben, 2002). The mean negative asynchrony and the negative relative phase angles confirmed that all participants but one tapped on average ahead of the metronome, anticipating the beat. Additionally, by looking at the resultant vector lengths, we also note that consistent synchronization was maintained throughout the task. Given these results, we can deduce that all subjects were engaged in the process of entraining their finger-taps to the auditory beats of the metronome.

As for the data captured by the EEG, our GED implementation was effective in extracting the target component maximally entrained to the rhythmic stimulus. Figure 3 provides a quality check for our source separation by combining the following three criteria at the group-level. The first, topography: the grand-average activation map of the selected component shows maximal activity in the left centroparietal cluster and in the left temporal electrodes. Such distribution strongly suggests the involvement of primary sensorimotor areas, given it is contralateral to the effector (i.e., the right hand). The same pattern was previously reported for movement-related SSEPs in the context of overt synchronized behavior (Nozaradan et al., 2015), and clearly differs from the frontocentral topography typical of auditory cortical responses in absence of movement (Nozaradan et al., 2012). Given our focus on sensorimotor dynamics underlying overt behavior, our spatial filter was constrained within the whole set of channels located behind the frontocentral line. Second, the power spectrum: a single major peak stands out at the metronome’s frequency, accompanied by harmonics whose power approximately follows a 1/f distribution. The dominance of the target frequency over the spectrum shows that the extracted component is effectively fine-tuned to the rhythmic stimulation. Third, the eigenspectrum: by sorting the eigenvalues in descending order, it is evident how the first one eigenvalue stands out over the rest of the spectrum. Such a condition is particularly desirable when the goal is to reduce the dimensionality of a multivariate dataset to one single component that satisfies a given criterion. The eigenvector associated with the highest eigenvalue could then be reliably used to weight the electrodes average, and reduce the dimensionality of the dataset to one entrained component.

Applying GED in the context of neural entrainment (Cohen and Gulbinaite, 2017) is an established method of optimizing source separation in this context, with avoidance of major drawbacks of electrode selection. To elaborate, we chose this approach instead of selecting a time series based on a single electrode or on a small cluster of electrodes in order to avoid subjective judgment to some degree. Despite this drawback, electrode selection is a rather common practice in the SSEP literature (Keitel et al., 2010; Andersen et al., 2011, 2012; Kashiwase et al., 2012; Rossion et al., 2012). In addition, with our spatial filter, we: (a) decrease the risk of attenuating the response in some subjects due to individual variability and (b) are not confounded by exposure to noise which might selectively affect a single channel. Although it is true that computing a non-weighted average over the whole scalp is sometimes proposed as a practice to avoid selection bias (e.g., see Nozaradan et al., 2016), the entrained response would be heavily attenuated by broadband components unrelated to the task. On the other hand, a weighted average oriented by spectral criteria would clearly overcome such issues. Most importantly, our methodology of GED application was optimal for single-trial analysis and provided us with a single time series whose time-course and dynamics could be further analyzed. Such time series represented the starting point of our pipeline towards the computation of the stability index (see Figure 2). It should also be noted that rhythmic motor acts such as finger-tapping (Moelants, 2002; McAuley, 2010) and walking (MacDougall and Moore, 2005) operate within the low delta frequency range (Morillon et al., 2019), which implies that long trials are needed to measure the dynamics of slower oscillatory components.

In order to validate our neural outcome measure of auditory-motor coupling, we ran correlation analyses with a set of behavioral measures of synchronization accuracy and stability (Moumdjian et al., 2018) in the context of a finger-tapping task to a metronome’s beats. The stability index exhibited moderate negative correlations with the relative phase angles, the mean asynchrony, and the resultant vector length. To explain our results, we first provide an explanation of the pattern we observed in the context of the stability of the frequency fluctuations, which are used to quantify the stability index. We observed less stability in the frequency fluctuations of the neural entrained component when the finger-taps were further away and with a wider distribution relative to the beats, as reported by the relative phase angles and resultant vector length, respectively. Conversely, when the finger taps were closer to and in anticipation of the beat, with a narrow distribution, we observed that the entrained neural component stabilized its frequency fluctuations. The results confirmed the hypothesis that these frequency fluctuations, as quantified by the stability index, correlated with the behavioral outcome measures of entrainment.

With our results, we also observed that the stability index was selectively correlated with measures of phase error correction mechanisms, and not with those of period error correction. This is consistent with the fact that the stability index was not correlated to the inter-beat deviations—which is a measure for quantifying tempo matching (Moumdjian et al., 2018). In turn, tempo matching is an outcome which describes error correction in a period. With the above explanations, our results are suggestive that the stability index quantifies neural entrainment, yet limited to corrections in phase. However, we do not rule out the possibility that we did not find any significant correlation due to the very low individual variability in inter-beat deviations, which resulted in a small slope of the regression line. The result indicates that participants were very accurate in matching the period of the metronome over the whole duration of the task.

The selectivity of these correlations further supports the relevance of temporal dynamics at the micro-timing scale. By picking up on the notion of “neural entrainment to the beat”, which is traditionally inferred from the Fourier coefficients of a “static” spectrum, we developed it towards a phase-based measure to make it sensitive to the temporal structure of the stimulus (Rajendran and Schnupp, 2019) and to behavioral dynamics. From our standpoint, in order to “entrain to the beat” a neural component should not only be tuned to the stimulus frequency, but it should dynamically attune depending on the ongoing entrained behavior. The stability index proposed in this context shows how frequency fluctuates over time as a function of the distance from in-phase synchronization (the phase angles and asynchrony) and consistency of the established relative-phase during the course of the task (resultant vector length). Previous work provided evidence on the correlation between cortical entrainment and overt sensorimotor synchronization (Nozaradan et al., 2016), recording brain activity by the means of the EEG during a passive listening task and subsequently performing the behavioral task. The authors detected entrained cortical activity on the frontocentral cluster of electrodes where auditory responses are typically detected, hypothesizing that SSEPs amplitudes would predict behavioral measures of overt entrainment. Interestingly enough, a dissociation emerged in the correlations between their measure of neural entrainment and behavioral accuracy, when compared to behavioral consistency. Specifically, the amplitude of SSEPs was related to mean asynchrony (accuracy) rather than to the resultant vector length (consistency), suggesting that the two are supported by distinct neural mechanisms when processing the beat of an auditory rhythm. In the scenario we proposed, with the goal of relating neural entrainment to the dynamics of overt behavior, we identified a lateralized component plausibly related to primary sensorimotor areas. The stability index computed from such component was related to both behavioral accuracy and consistency.

Our finding is arguably not in contradiction with previous evidence, but rather complementary.

One may argue that the correlation we found between the stability index and the resultant vector length could be spurious, a sort of epiphenomenon entirely explainable by afferent proprioceptive feedback. Following this argument, stable rhythmic behavior could produce steady responses in primary somatosensory areas (Piitulainen et al., 2013; Bourguignon et al., 2015). Our task cannot exclude the possibility that such afferent components lead to a spurious correlation between the stability index and resultant vector length, which quantifies behavioral consistency. Nevertheless, such interpretation cannot explain our crucial finding that the stability index also correlated with behavioral accuracy. To elaborate, a more stable entrained component was associated with smaller synchronization errors, as quantified by measures of asynchrony and relative phase. In this context, these are behavioral indicators of error correction. Since we showed that a more stable entrained component correlated with smaller error relative to the beat, we propose that the stability index is not merely determined by proprioceptive feedback. We thus argue that our results rather align with evidence that motor cortices play a critical role in supporting auditory perception and prediction (Fujioka et al., 2012; Morillon and Baillet, 2017; Assaneo et al., 2021). In addition, within the limits of our auditory-motor task, we pick up on the notion of active sampling (Morillon et al., 2019), to propose that entrainment dynamics driven by the motor system seem to play an active role in the predictive mechanism of error minimization underpinning auditory-motor coupling (Vuust and Witek, 2014). However, with the current experimental design, we cannot rule out that distinct motor, sensory and cognitive processes were to some extent mixed in the entrained component. This represents an important limitation of the present study. A finer disentanglement of the neural processes underlying entrainment should be addressed by future work, with dedicated experimental designs.

With our work, we thus contributed methodologically to the investigation of neural entrainment. Our method consists of extracting the oscillatory component in the EEG signal which is maximally entrained to a rhythmic auditory stimulus and subsequently quantifying the stability of fluctuations over time. The impact of this contribution has valuable prospects within the domain of neurological rehabilitation. In previous work, we have investigated motor and auditory entrainment in participants with multiple sclerosis and healthy controls. Specifically, behavioral time series were analyzed by means of detrended fluctuation analysis (DFA; Moumdjian et al., 2020). Differences in gait dynamics were attributed to the process of error-correction minimization, which are required to dynamically interact with continuous and discrete auditory structures (Moumdjian et al., 2020) typically present in music and metronomes, respectively. Although clinically relevant, complementing such studies with neural outcome measures such as the stability index would allow to explain the process of error-correction minimization further, at the level of neural dynamics. Such a prospect has a strong indication to optimize the individualized rehabilitation procedure.

In conclusion, our approach can be used for better understanding the dynamics of an entrained system over time. While the stability index provides a global neural outcome measure correlated with the overall synchronization performance, the instantaneous frequency time series can offer a more fine-grained picture of the dynamics of neural entrainment. Neural and behavioral measurements can be complemented within a comparative setting between healthy population and neuropathological models, offering the possibility to dissociate neural mechanisms based on a mapping of selective lesions. Such neuropathological models can be recruited through studies conducted on participants with neurological diseases, where components of cognitive, motor or, perceptual functions can be isolated. For instance, cerebellar lesions cover particular interest given the role of the cerebellum in encoding the timing of events at the micro-timing scale (Ivry et al., 1988; Ivry and Keele, 1989; Ivry and Schlerf, 2008), and given that their neural entrainment to auditory rhythms is selectively compromised at faster tempi (Nozaradan et al., 2017). Respectively, this unfolding of observations would expand the knowledge of the complex dynamic interaction when entraining motor and auditory systems to one another. In turn, it would pave ways towards the development of state-of-the-art approaches within the domain of neurological rehabilitation.

## Data Availability Statement

The datasets presented in this article are not readily available because the raw data generated for the current study (from the EEG recordings) are a part of a bigger experimental design. Given software restrictions, we are not able to disintangle the applicable dataset from this study at the level of the raw recordings. However, it is possible for us to provide pre-processed data of the experimental condition used in this study upon request. Requests to access the datasets should be directed to mattia.rosso@ugent.be.

## Ethics Statement

The studies involving human participants were reviewed and approved by Ethics Committee of Ghent University (Faculty of Arts and Philosophy). The patients/participants provided their written informed consent to participate in this study.

## Author Contributions

MR was responsible for study conceptualization, data collection, processing and analysis, and writing of the manuscript. ML was responsible for data interpretation and manuscript writing. LM was responsible for study conceptualization, data interpretation, and writing of the manuscript. All authors contributed to the article and approved the submitted version.

## Conflict of Interest

The authors declare that the research was conducted in the absence of any commercial or financial relationships that could be construed as a potential conflict of interest.

## Supplementary Material

SUPPLEMENTARY FIGURE 1 Narrow-band filter kernel for GED. The figure shows the wavelet kernel in the frequency domain, centered on the target frequency of 1.65 Hz. In order to set the width of the Gaussian, we opted for a filter that would allow some extent of fluctuations around the centered frequency, without overlapping with the high-pass band filter (cut-off = 1 Hz). We found an optimal trade-off by setting the Gaussian width at half-maximum at 0.3 Hz. It is important to note that the correlations with the behavioral measures reported in the present work are invariant to such parameters. However, one should be aware that the scale of the stability index is inversely proportional to the width of the narrow-band filter. This is due to the fact that a wider filter allows for wider frequency fluctuations. Finally, the scale of the stability index is also affected by the filter shape. It was previously proposed that symmetric plateau-shaped filters should be preferred over a Gaussian when investigating frequency shifts, since the latter is biased towards the center frequency (Cohen, 2014). However, we found that the correlations are invariant when comparing plateau-shaped FIR and Gaussian-shaped, as long as both are symmetrical. After verifying the invariance of the results, we opted for a Gaussian filter for the sake of parsimony and replicability: given that the center frequency is constrained by the rate of the stimulation, the width is the only parameter to be defined by the data analyst.

SUPPLEMENTARY FIGURE 2 Component #2. As it can be evicted by the right-most plot, the second eigenvalue detaches to some extent in the eigenspectrum. We present here the activation map and the power spectrum (normalized to signal-to-noise ratio) of the component associated with the second eigenvalue. The spectral profile is clearly characterized by dominant peaks at entrained frequency and harmonics, which is to be expected given the spectral criterion adopted for the GED. It is noteworthy that the signal-to-noise ratio is considerably lower as compared to the first component. Critically, no meaningful pattern emerged from the topographical activation map.

SUPPLEMENTARY FIGURE 3 Component #2 (correlations with behavioral outcome measures). The stability index was computed from the second component, and its correlations with the behavioral outcome measures were tested. Correlations were considerably weaker than the ones for the component associated with the highest eigenvalue, as quantified by the Spearman correlation coefficients. Results are showed in the original scale and transformed to ranks. This evidence suggests that the first component alone is related to neural entrainment in the context of the experimental task. Acknowledging that we still cannot completely rule out the merging of different neural processes across components, the approach hereby proposed has proved to be valid in extracting one single component related to auditory-motor coupling.
