# Supplementary figures and images for: Neural Entrainment Meets Behavior: The Stability Index as a Neural Outcome Measure of Auditory-Motor Coupling
Source: Front Hum Neurosci. 2021 Jun 9;15:668918. doi: 10.3389/fnhum.2021.668918 (PMC8219856; doi:10.3389/fnhum.2021.668918)

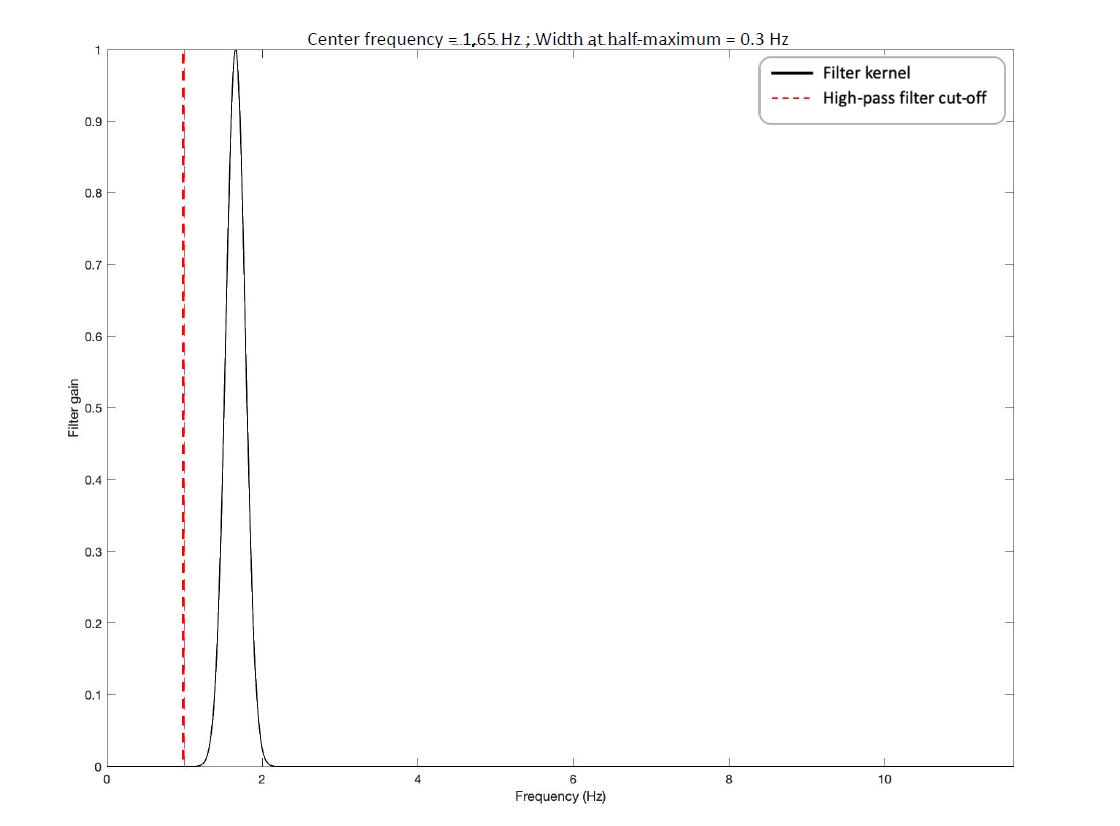

Supplement: SUPPLEMENTARY FIGURE 1 — Narrow-band filter kernel for GED. The figure shows the wavelet kernel in the frequency domain, centered on the target frequency of 1.65 Hz. In order to set the width of the Gaussian, we opted for a filter that would allow some extent of fluctuations around the centered frequency, without overlapping with the high-pass band filter (cut-off = 1 Hz). We found an optimal trade-off by setting the Gaussian width at half-maximum at 0.3 Hz. It is important to note that the correlations with the behavioral measures reported in the present work are invariant to such parameters. However, one should be aware that the scale of the stability index is inversely proportional to the width of the narrow-band filter. This is due to the fact that a wider filter allows for wider frequency fluctuations. Finally, the scale of the stability index is also affected by the filter shape. It was previously proposed that symmetric plateau-shaped filters should be preferred over a Gaussian when investigating frequency shifts, since the latter is biased towards the center frequency (Cohen, 2014). However, we found that the correlations are invariant when comparing plateau-shaped FIR and Gaussian-shaped, as long as both are symmetrical. After verifying the invariance of the results, we opted for a Gaussian filter for the sake of parsimony and replicability: given that the center frequency is constrained by the rate of the stimulation, the width is the only parameter to be defined by the data analyst. [file Image_1.jpeg]

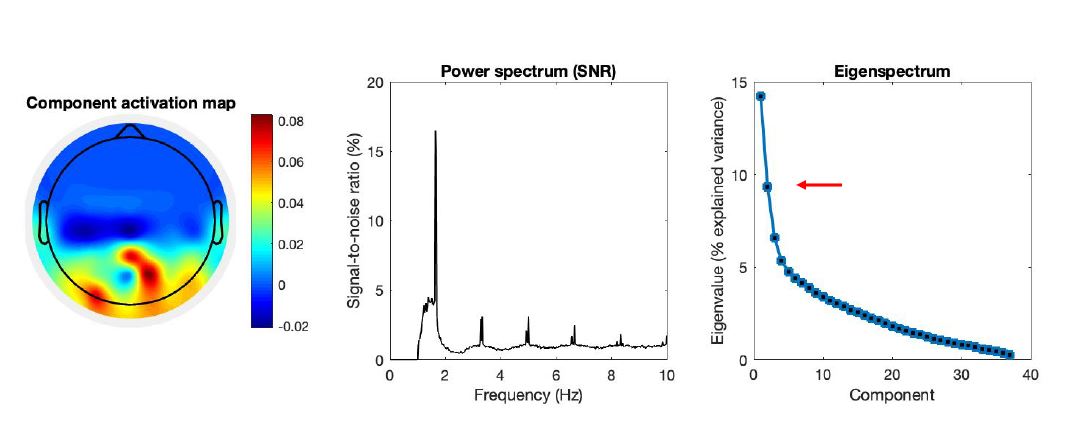

Supplement: SUPPLEMENTARY FIGURE 2 — Component #2. As it can be evicted by the right-most plot, the second eigenvalue detaches to some extent in the eigenspectrum. We present here the activation map and the power spectrum (normalized to signal-to-noise ratio) of the component associated with the second eigenvalue. The spectral profile is clearly characterized by dominant peaks at entrained frequency and harmonics, which is to be expected given the spectral criterion adopted for the GED. It is noteworthy that the signal-to-noise ratio is considerably lower as compared to the first component. Critically, no meaningful pattern emerged from the topographical activation map. [file Image_2.jpeg]

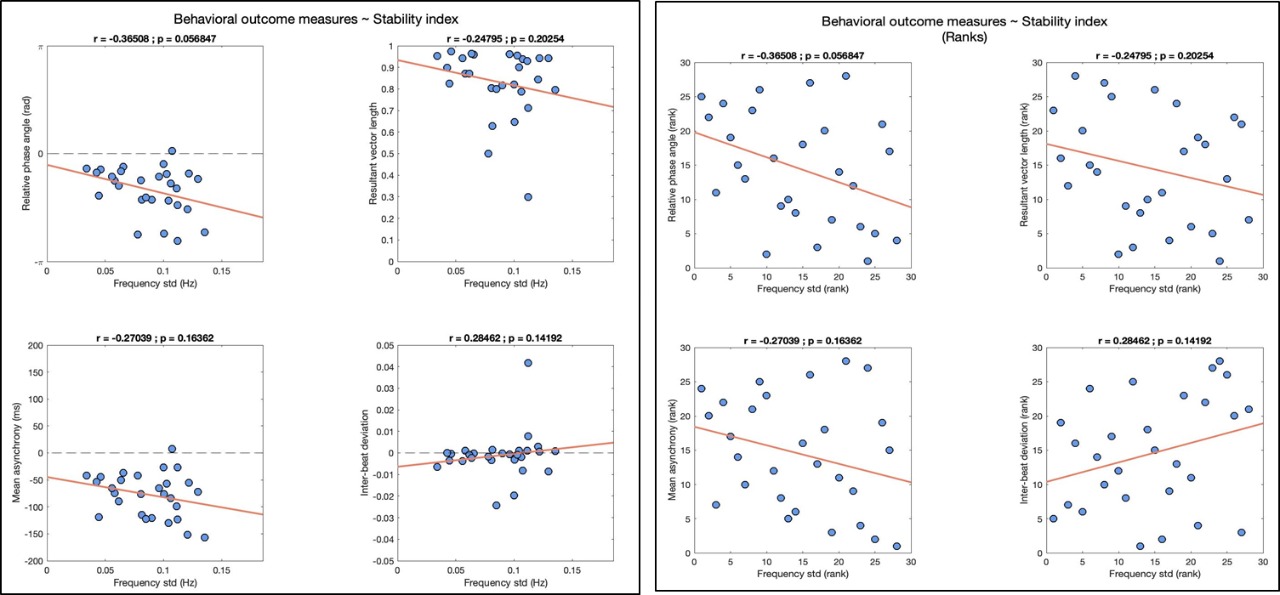

Supplement: SUPPLEMENTARY FIGURE 3 — Component #2 (correlations with behavioral outcome measures). The stability index was computed from the second component, and its correlations with the behavioral outcome measures were tested. Correlations were considerably weaker than the ones for the component associated with the highest eigenvalue, as quantified by the Spearman correlation coefficients. Results are showed in the original scale and transformed to ranks. This evidence suggests that the first component alone is related to neural entrainment in the context of the experimental task. Acknowledging that we still cannot completely rule out the merging of different neural processes across components, the approach hereby proposed has proved to be valid in extracting one single component related to auditory-motor coupling. [file Image_3.jpeg]
